# One Health integrated strategies for sustainable control of *Opisthorchis viverrini* infections in rural endemic areas of Thailand

**DOI:** 10.1186/s40249-025-01315-7

**Published:** 2025-06-03

**Authors:** Suksanti Prakobwong, Lakhanawan Charoensuk, Suwit Chaipibool, Kacha Chedtabud, Umawadee Laothong, Apiporn T. Suwannatrai, David Blair, Somchai Pinlaor

**Affiliations:** 1 Department of Biology, Faculty of Science, Udon Thani Rajabhat University, Udon Thani, Thailand; 2 The Eco-Health and Parasitology Research Unit, Faculty of Science, Udon Thani Rajabhat University, Udon Thani, 41000 Thailand; 3 Department of Clinical Pathology, Faculty of Medicine Vajira Hospital, Navamindradhiraj University, Bangkok, 10300 Thailand; 4 Nong-Sang Hospital of Health Promotion, Nong Wua Sor District, Udon Thani, 41360 Thailand; 5 Department of Geoinformatics for Development, Faculty of Humanities and Social Sciences, Udon Thani Rajabhat University, Udon Thani, 41000 Thailand; 6 Department of Community Health, Faculty of Public Health, Mahidol University, Bangkok, 10400 Thailand; 7 Department of Parasitology, Faculty of Medicine, Khon Kaen University, Khon Kaen, 40002 Thailand; 8 College of Science and Engineering, James Cook University, Townsville, QLD 4811 Australia; 9 Cholangiocarcinoma Research Institute, Faculty of Medicine, Khon Kaen University, Khon Kaen, 40002 Thailand

**Keywords:** *Opisthorchis viverrini*, One Health, Environmental control, Sustainable prevention, Intervention method

## Abstract

**Background:**

Opisthorchiasis, caused by *Opisthorchis viverrini*, poses a significant health risk in northeastern Thailand, increasing the prevalence of cholangiocarcinoma. This study implemented a One Health integrated strategy, targeting human, animal, and environmental factors to reduce *O. viverrini* prevalence and transmission in an endemic region.

**Methods:**

The study was conducted from 2016 to 2022 in the Huay Luang Reservoir area, Udon Thani Province, Thailand and enrolled 5412 participants. Annual stool examinations were conducted and participants found to be infected with *O. viverrini* received anthelmintic treatments. Other intervention methods included health education, snail control, veterinary care, sanitation improvements, training of health volunteers, creating a learning center and liver fluke-free fish production. Annual data on prevalence, infection intensity, and reinfection rates were collected. Student’s *t*-test, one-way ANOVA, Chi-square test, or Fisher’s exact test were used to compare data across the study years, with statistical significance set at *P* < 0.05.

**Results:**

The One Health strategy significantly reduced *O. viverrini* prevalence in humans from 14.1% in 2016 to 0.9% in 2022, with *O. viverrini*-egg intensity decreasing from 76.9 to 25.5 eggs per gram (EPG) (*P* < 0.001). Reinfection rates decreased significantly from 17.4% in 2016 to 9.7% in 2022 following the implementation of the program (*P* = 0.003). Among reservoir hosts, infections in dogs and cats significantly decreased from 21.3% to 3.8% (*P* < 0.001). In cyprinoid fish, metacercarial prevalence significantly decreased from 21.9% to 2.2% (*P* < 0.001). Awareness of transmission routes rose from 45.1% to 82.6%, and raw fish consumption decreased from 52.4% to 12.3%. Biological control reduced *Bithynia* snail densities from 30 to under 5 snails/m^2^, while sanitation interventions increased toilet use from 31.7% to 87.1%. A local fish-processing enterprise enhanced food safety and income. Health volunteers engaged 94% of households, and a learning center trained 250 individuals and hosted site visits.

**Conclusions:**

The One Health strategy effectively and sustainably limited *O. viverrini* infections and reinfections, demonstrating the potential of One Health as a model for zoonotic parasite control in other endemic areas.

**Supplementary Information:**

The online version contains supplementary material available at 10.1186/s40249-025-01315-7.

## Background

Opisthorchiasis, caused by the liver fluke *Opisthorchis viverrini*, is a significant public health concern in Southeast Asia [1]. In Thailand, the highest prevalence occurs in northeastern provinces due to the local habit of eating raw or undercooked freshwater fish containing infective metacercariae [2]. Despite considerable effort, prevention and control of the disease remain inadequate, sustaining the transmission of liver fluke and risk of cholangiocarcinoma (CCA) in endemic areas [3]. The life cycle of *O. viverrini* is sustained by complex interactions between humans, animals, and the environment. *Bithynia* snails and cyprinoid fish serve as first and second intermediate hosts, respectively [4]. Reservoir hosts, such as dogs and cats, may contribute to transmission through fecal contamination of water sources [5]. Environmental contamination of aquaculture systems perpetuates the parasite’s life cycle [6, 7]. These interconnected factors highlight the need for holistic approaches that address multiple dimensions of the disease simultaneously.

The One Health framework provides an integrated and interdisciplinary approach to addressing zoonotic and environmentally driven diseases [8, 9]. This framework is particularly relevant for controlling *O. viverrini*, as it enables interventions targeting all stages of the parasite’s life cycle [10]. By focusing on human health through education and behavioral change, managing zoonotic transmission in animal reservoirs, and improving environmental sanitation, One Health offers a comprehensive strategy for controlling opisthorchiasis. Previous studies have demonstrated the effectiveness of One Health approaches in controlling parasitic diseases, highlighting the importance of community involvement in sustaining these interventions [9]. Integrated strategies combining veterinary interventions, snail control, and health education have significantly reduced prevalence of *O. viverrini* [11]. Despite these efforts, the long-term sustainability of this model remains uncertain due to the persistent challenge of reinfection [12, 13].

This study was conducted from 2016 to 2022 in the Huay Luang Reservoir area of Udon Thani Province, Thailand, an endemic hotspot where previous surveys, constituting a pilot study for this work, reported *O. viverrini* prevalence of 31.5% [14]. The Huay Luang Reservoir, located in the upper northeastern region of Thailand, serves more than a million residents who depend on this water basin for irrigation, fisheries, agriculture and their daily needs. This extensive usage significantly influences the transmission and prevalence of opisthorchiasis. The primary aim of the study was to reduce *O. viverrini* infections and to sustainably maintain the reduction. The first step was to establish baseline data on the prevalence and intensity of infections in humans, reservoir hosts, and environmental sources. The One Health strategy employed in this study integrated health-education campaigns, biological control of snail hosts, veterinary interventions for dogs and cats (reservoir hosts), and environmental sanitation improvements. Community engagement was developed to control and prevent opisthorchiasis in a sustainable manner, with the additional aim of promoting sustainable economic practices through liver fluke-free fish production by local enterprises. This study provides a comprehensive analysis of a One Health integrated strategy, highlighting its potential as a sustainable and replicable model for managing *O. viverrini* infections and other zoonotic diseases.

## Methods

### The protocol of the study and participants

**Fig. 1:**
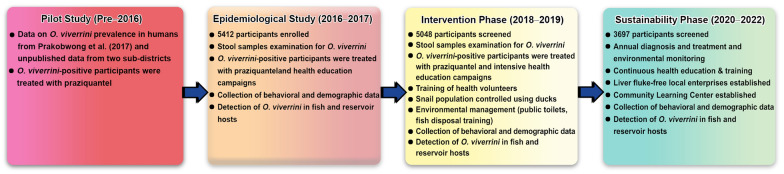
Flow diagram outlining the elements of the study. Annual monitoring included assessments of prevalence and infection intensity (EPG), risk-factor surveys using questionnaires, estimates of fish metacercarial density, data on CCA cases, and GIS mapping of infection distribution

This longitudinal observational study was conducted in an endemic region in Udon Thani Province involving 24 villages in 5 Sub-districts surrounding the Huay Luang Reservoir. This reservoir has a capacity of approximately 130 million cubic meters, and its downstream river basin, spanning 2000 square kilometers in Northeast Thailand. Beginning in 2016 and extending through July 2022, the study enrolled 5412 participants aged 7 to 84 years. The study plan included annual diagnosis and treatment, disease prevention via health-education programs, and support for local income-generating activities to foster long-term behavioral changes. The research was conducted in distinct phases. The initial epidemiological survey phase, conducted between 2016 and 2017, collected baseline data on *O. viverrini* prevalence. Treatment of parasites with anthelmintics and health-education initiatives were implemented in the communities during this phase. The intervention phase, conducted in 2018 and 2019 with the same participants, targeted all study communities through diagnosis of opisthorchiasis, anthelmintic administration, health education, sanitation and hygiene improvements, and biological control of snail populations using ducks. The sustainability phase, spanning 2020 to 2022, focused on maintaining progress in highly endemic communities. Flow diagram outlining the elements of the study is shown in Fig. 1. Images depicting various activities conducted during this research are provided in the supplementary figure.

The effect of the implemented strategies was assessed annually. A questionnaire was designed to analyze risk factors and inform the ongoing control program. Fecal samples from participants and mammalian reservoir hosts as well as cyprinoid fish from local sources were annually collected to investigate the prevalence and intensity of *O. viverrini* infection, as well as the reinfection rate in participants.

The study was approved by the Human Research Ethics Committee of the Ministry of Health (HE6002) and the Human Research Ethics Committee of Udon Thani Rajabhat University (HEUCD.03/2017, HEUCD.10/2019, and HEUCD.75/2021). The study in animals was approved by the Animal Ethics Committee of Udon Thani Rajabhat University (AREC.UDRU.01/2016 and AREC.UDRU.03/2022).

### Pilot study

Data collected prior to 2016 and reported by Prakobwong et al. in 2017 [14] constituted a pilot study. The data included the number of human opisthisthorchiasis cases and the prevalence of *O. viverrini* infection, incorporating unpublished data from two sub-districts and previously reported findings from three sub-districts. Individuals infected with *O. viverrini* were treated with praziquantel.

### Epidemiological study and initial health education (2016−2017)

#### Comprehensive diagnosis and treatment for *O. viverrini* infections and health education

The study included 5412 participants selected through stratified random sampling to ensure representation across various age groups, occupations and both genders. Baseline data on demographic characteristics, dietary habits, and knowledge of opisthorchiasis were collected using structured questionnaires. Stool samples from all participants were examined for *O. viverrini* eggs using the one-step formalin ethyl-acetate concentration technique (one-step FECT) as previously described [15]. Initial health-education campaigns were conducted to raise awareness about the risks of consuming raw or undercooked fish and to promote safe eating practices. Educational materials, including posters and community meetings, were used to disseminate information to all residents of the study area about opisthorchiasis and its prevention. Volunteers were recruited in each village and engaged with their communities to take responsibility for household education and awareness.

A survey of infective-stage *O. viverrini* metacercariae was done on 420 cyprinoid fish during this phase (2016–2017). Fish were digested using 0.25% pepsin, as previously described [16]. Infection in dogs and cats (451 animals total), which serve as reservoir hosts in the community, was diagnosed using the one-step FECT. Infected participants and reservoir hosts received appropriate treatment for *O. viverrini* infections. Praziquantel at a single dose of 40 mg/kg body weight was administered to individuals who tested positive for *O. viverrini* eggs. Individuals with stool samples yielding egg counts exceeding 500 eggs per gram (EPG) of feces were randomly selected for re-examination 7–14 days later to confirm the success of treatment. Treatment of infected dogs and cats followed the same protocol.

### Intervention phase (2018–2019)

#### Screening for *O. viverrini* infection in participants

Stool samples were collected from 5048 participants and tested for *O. viverrini* eggs. Infected individuals were treated with praziquantel as described above to reduce the parasite burden within the community.

#### Training of health volunteers and intensive health education

A network of health volunteers was established to support opisthorchiasis prevention efforts within the community. Volunteers were recruited from non-*O. viverrini* infected participants and received training in basic health education and monitoring, enabling them to act as facilitators for disease control. At least 10 volunteers were recruited in each village, with each volunteer responsible for 10–15 households. Preventive health education was conducted once/year using a paper-based approach, targeting schoolchildren in schools, *O. viverrini*-infected participants, and high-risk groups in their villages. Educational materials included pamphlets, posters, community meetings, and study plans. Students were provided with learning resources to support their studies. Demonstrations of safe food preparation reinforced these messages, ensuring widespread awareness and encouraging active community participation.

#### Biological control of snail population

The density of *Bithynia* snails was managed using domestic ducks as a biological control measure. Ducks were reared by participants’ households and deployed at the rate of five ducks per 100 m^2^ of water surface. Snail populations in privately owned swamps, ponds and rice fields were subjected to this control approach throughout the study. Interventions in the main reservoir were conducted once during each study phase using 5000 ducks. Snail density in two randomly selected 100 m^2^ areas of each privately owned swamp and pond was monitored twice a year, while snail density in the main reservoir was monitored both before and after duck usage.

#### Environmental management

A total of 317 dogs and cats, primarily dogs, in the community were tested using one-step FECT and treated for any liver fluke infections found. Those positive for *O. viverrini* eggs were treated with a single dose of 40 mg/kg body weight of praziquantel by veterinary services volunteers. Fishermen were educated on proper disposal methods to avoid discarding decayed fish around the reservoir. Additionally, we built 10 public toilets with septic systems (2 × 3 m) in rice fields and near the reservoir for farmers and fisherman to use. The frequency and number of individuals utilizing these toilets were collected via annual questionnaires, visits by health volunteers, and records of participants. Cyprinoid fish (*n* = 452) were examined for *O. viverrini* metacercariae.

### Sustainability phase (2020−2022)

#### Determination of *O. viverrini* infection in hosts and anthelminthic treatment

Stool samples were collected from 3697 individuals who had participated in earlier phases and 320 reservoir host animals were examined for *O. viverrini* eggs. Infected individuals and animals were treated with praziquantel at a dose of 40 mg/kg body weight. Cyprinoid fish (*n* = 417) were examined for *O. viverrini* metacercariae.

#### Continuing health education and training programs

Ongoing health-education initiatives were implemented to maintain awareness and knowledge about opisthorchiasis and cholangiocarcinoma. Community health volunteers were trained to deliver educational messages and support behavior-change efforts. Training programs enhanced the diagnostic skills of health officials, equipping them to identify and manage opisthorchiasis cases effectively. Workshops and practical training sessions emphasized advanced diagnostic techniques and updated treatment protocols to ensure accurate and efficient case management.

#### Establishment of liver fluke-free local fish-processing enterprises

Local enterprises involved in on-land fish farming and food production from farms and the reservoir were established to adopt liver fluke-free practices. Processing systems included raw material reception, production, and packaging processes. Raw fish was assessed for trematode metacercariae, and *Escherichia coli* contamination was evaluated and prevented. Certification programs were introduced to promote and recognize businesses that adhere to safe and hygienic practices, reducing the risk of infection. The quantity of raw fish, fish products, costs, income and profits were recorded each year.

#### Community learning center

A community learning center dedicated to opisthorchiasis prevention and control was established to serve as a resource hub for information, training, and outreach activities.

The center provided educational materials, hosted training sessions, and facilitated community engagement in prevention efforts.

### Assessment of program

The effectiveness of the implemented strategies was assessed annually at the beginning of each study year based on the number of human opisthorchiasis cases, prevalence, and the intensity of *O. viverrini* infection (measured as EPG). Reinfection rates in the communities from 2016 to 2022 were analyzed using data from this study and the pilot study [14] and were assessed through follow-up stool sample analysis one year after treatment of the individuals. A questionnaire was developed to assess risk factors and guide the implementation of interventions across different phases. Numbers of metacercariae in infected fish were assessed.

### Statistical analysis

Data were analyzed using SPSS Version 23 (SPSS Inc., Chicago, IL, USA). Continuous variables with a normal distribution were presented as mean ± standard deviation (*SD*), while non-normal variables were reported as median with interquartile range. Quantitative parameters were compared across years using Student’s *t*-test and one-way ANOVA, while the Chi-square or Fisher’s exact test was applied for qualitative data. Independent comparisons were used to evaluate EPG counts in humans and metacercarial density in fish across study phases. Statistical significance was set at *P* < 0.05. Data on the number of CCA cases were obtained at the end of each year from hospitals in the study area. The spatial distribution and levels of EPG in households were mapped using administrative boundary data. Publicly available datasets from the Geo-Informatics and Space Technology Development Agency (GISTDA) and Google Maps were utilized, and the analysis was conducted using ArcGIS software (ESRI, Redlands, CA).

## Results

### Impact of the project on the prevalence and intensity of *O. viverrini* infections in humans

**Fig. 2:**
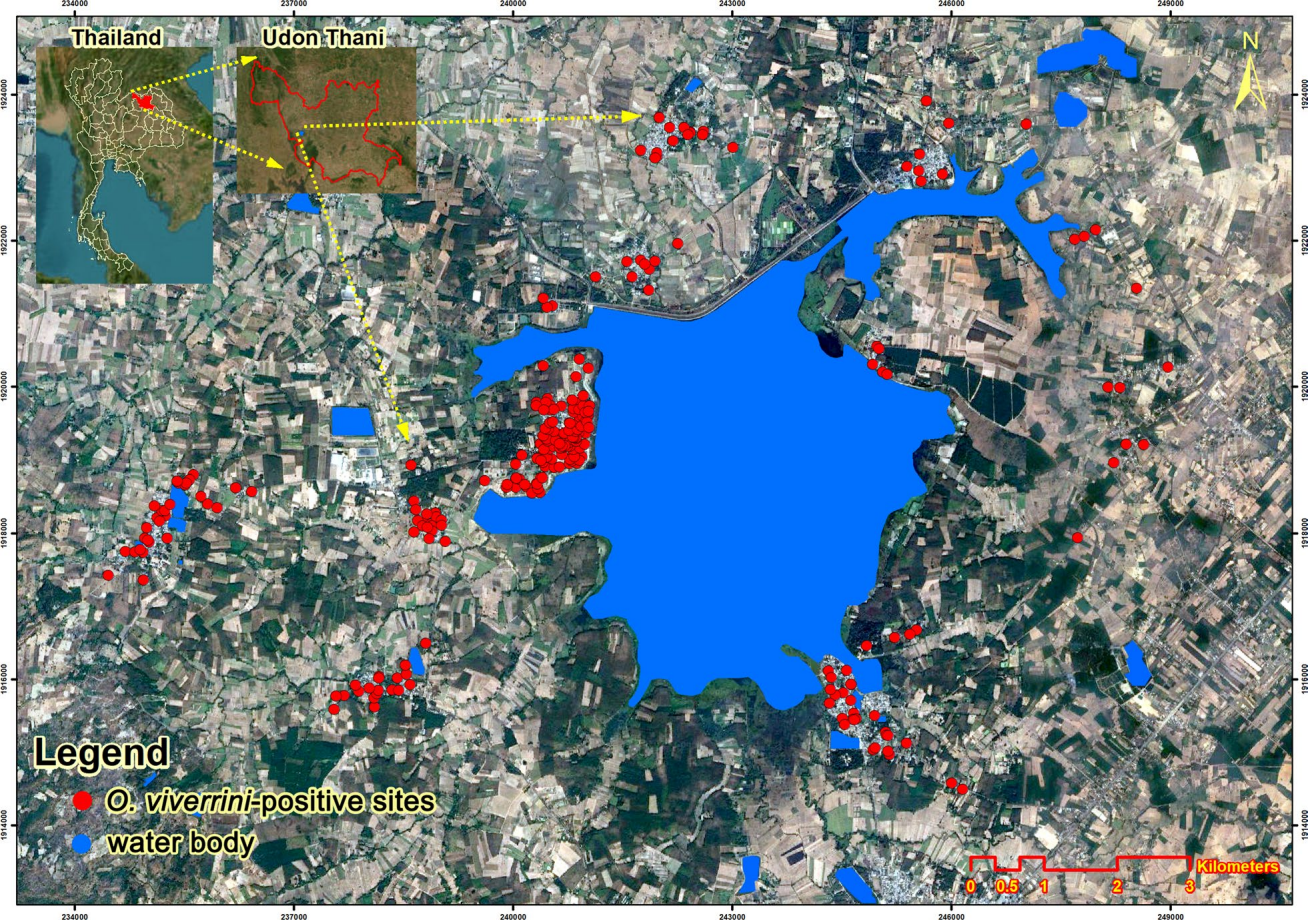
Distribution of households of *Opisthorchis viverrini*-infected participants in year 2022. The map includes the water sources analyzed in this study

**Fig. 3:**
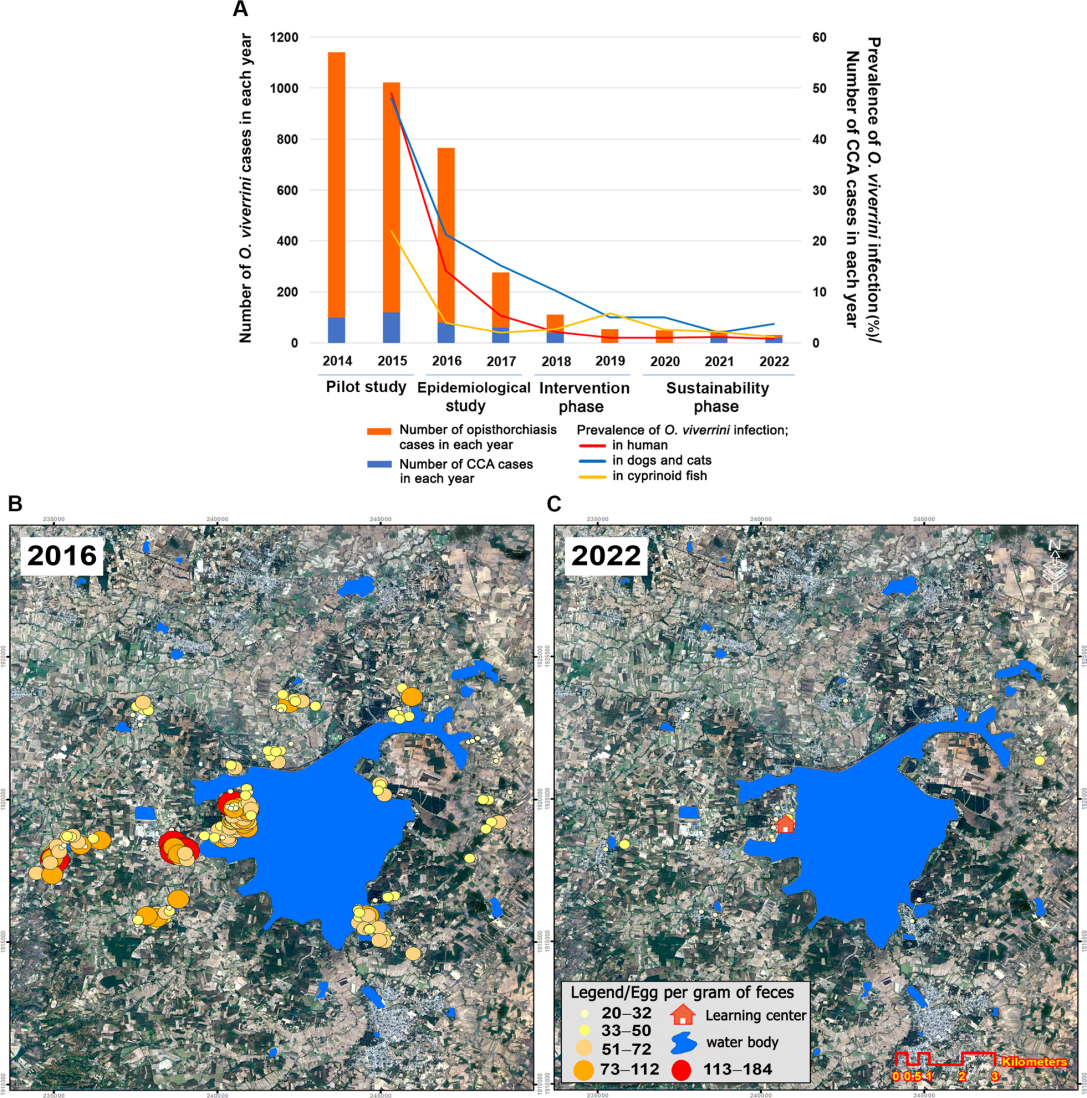
Number of human opisthorchiasis cases and prevalence of *Opisthorchis viverrini* infection in hosts observed in this study, and references to the number of cholangiocarcinoma cases for each year (**A**). Reduction in the intensity of *O. viverrini* infection and the number of infected households in the study areas (**B** and **C**). The location of the Learning Center in the study area is included

The baseline prevalence of opisthorchiasis during the epidemiological phase (2016**−**2017) was 14.1% (765 out of 5412 participants) (Table 1). The distribution of *O. viverrini*-positive households in 2016 is shown in Fig. 2. Following the intervention, positive cases significantly decreased to 1.1% in 2019 and further declined to 0.9% during the sustainability phase in 2022 (*P* < 0.001). This reduction was consistent across the 31–40-year age group, education levels, and occupations, with notable improvements among primary school graduates and farmers, who initially had the highest prevalence. Infection intensity, measured as EPG of feces, significantly declined from 76.9 in 2016 to 37.3 in 2019 and 25.5 in 2022 (*P* < 0.001) (Table 2). Reduction in prevalence in hosts is presented in Fig. 3A, and spatial representation of the reduction in prevalence and infection intensity (using EPG as a proxy) among humans is presented in Fig. 3B** − **C.

**Table 1:** Prevalence of *Opisthorchis viverrini* infection in participants, with behavioral and demographic correlates

Characteristics of participants	Epidemiological study (year 2016)		Intervention phase (year 2019)		Sustainability phase (year 2022)		*P*-value
	*n*	Number infected with* O. viverrini* (%)	*n*	Number infected with* O. viverrini* (%)	*n*	Number infected with* O. viverrini* (%)	
Age (years)							
< 20	124	12 (9.7%)	96	1 (1.0%)	23	1 (4.3%)	< 0.001*
20**−**29	1547	145 (9.4%)	1043	14 (1.3%)	645	8 (1.2%)	
30**−**39	3246	**570 (17.6%)**	3055	22 (0.7%)	2356	18 (0.8%)	
40**−**49	136	9 (6.6%)	341	9 (2.6%)	295	3 (1.0%)	
> 50	359	29 (8.1%)	413	8 (1.9%)	378	2 (0.5%)	
Sex							
Female	3056	438 (14.3%)	2753	35 (1.3%)	1978	15 (0.8%)	0.263
Male	2356	327 (13.9%)	2195	19 (0.9%)	1719	17 (1.0%)	
Occupation							
Fisherman	481	114 (23.7%)	412	14 (3.4%)	376	3 (0.8%)	0.128
Farmer	4045	566 (14.0%)	3710	30 (0.8%)	2687	24 (0.9%)	
Government	425	51 (12.0%)	404	6 (1.5%)	359	3 (0.8%)	
Others	461	34 (7.4%)	422	4 (0.9%)	275	2 (0.7%)	
Education							
Primary school	2845	**540 (19.0%)**	2576	27 (1.0%)	1976	17 (0.9%)	< 0.001*
Secondary school	1642	189 (11.5%)	1558	13 (0.8%)	1071	8 (0.7%)	
Tertiary level	925	36 (3.9%)	814	14 (1.7%)	650	7 (1.1%)	
History of taking anthelminthic drug in previous 5 years							
Yes	892	125 (14.0%)	677	7 (1.0%)	546	6 (1.1%)	0.748
No	4520	640 (14.2%)	4,271	47 (1.1%)	3151	26 (0.8%)	
The annual frequency of consuming raw fish							
Never	2574	162 (6.3%)	2374	28 (1.2%)	2841	16 (0.6%)	< 0.001*
Occasionally	1974	268 (13.6%)	1802	14 (0.8%)	566	12 (2.1%)	
More than three times	864	**335 (38.8%)**	772	12 (1.6%)	290	4 (1.4%)	
History of relative dying from CCA							
Yes	647	96 (14.8%)	604	10 (1.7%)	395	4 (1.0%)	0.448
No	4765	669 (14.0%)	4344	44 (1.0%)	3302	28 (0.8%)	
Totals	5412	765 (14.1%)	4948	54 (1.1%)	3697	32 (0.9%)	

^*^ *P*-value < 0.05

Chi-squared test or Fisher’s-exact test was used to compare proportions or percentages across the three phases of the study

Bold numbers indicate statistically significant highest values in positive cases or intensity across the seven years

**Table 2:** Prevalence of *Opisthorchis viverrini* infection in hosts during 2016**−**2022

Phases	Year	Human			Dogs/Cats			Cyprinoid fish		
		*n*	Positive (%)	Average EPG (range)	*n*	Positive (%)	Average EPG (range)	*n*	Positive (%)	Average metacercariae/infected fish (range)
Epidemiological study	2016	5412	**765 (14.1%)**	**76.9 (18−576)**	451	**96 (21.3%)**	**43.9 (22−136)**	420	**92 (21.9%)**	**6.0 (2−26)**
	2017	5128	276 (5.4%)	41.3 (18**−**68)	342	52 (15.2%)	32.5 (18**−**56)	350	14 (4.0%)	4.1 (2**−**8)
Intervention phase	2018	5091	111 (2.2%)	27.5 (18**−**38)	340	35 (10.3%)	23.8 (18**−**42)	248	5 (2.0%)	3.4 (2**−**6)
	2019	5048	54 (1.1%)	37.3 (18**−**86)	317	16 (5.0%)	32.5 (22**−**44)	452	26 (5.8%)	4.3 (2**−**10)
Sustainability phase	2020	4821	50 (1.0%)	28.8 (18**−**46)	358	18 (5.0%)	22.8 (18**−**34)	344	7 (2.0%)	2.9 (2**−**4)
	2021	4035	48 (1.2%)	34.4 (18**−**38)	341	7 (2.1%)	22.9 (18**−**32)	340	9 (2.6%)	2.8 (2**−**4)
	2022	3697	32 (0.9%)	25.5 (18**−**48)	320	12 (3.8%)	27.1 (22**−**34)	417	9 (2.2%)	4.3 (2**−**9)
*P-value*			< 0.001^a^	< 0.001^b^		< 0.001^a^	< 0.001^b^		0.001^a^	0.003^b^

^a^ Chi-squared test comparing the annual proportion of positive cases across the seven years of the study

^b^ One-way ANOVA was used to compare the annual mean intensity across the seven years of the study

Bold numbers indicate statistically significant highest values in positive cases or intensity across the seven years. *EPG* Eggs per gram

### Infection rates in reservoir hosts and cyprinoid fish

The prevalence of opisthorchiasis in dogs and cats was 21.3% (96 out of 451 animals) during the initial phase in 2016 (Table 2). A statistically significant decline was observed, with prevalence decreasing to 5.0% in 2019 and further to 3.8% during the sustainability phase in 2022 (*P* < 0.001). Infection intensity in these animals, measured as EPG of feces, also significantly decreased from 43.9 in 2016 to 27.1 in 2022 (*P* < 0.001). Among cyprinoid fish, the prevalence of infection with metacercariae showed substantial reductions, from 21.9% in 2016 to 2.2% in 2022 (*P* < 0.001). This trend was consistent across both rice fields and reservoir, with prevalence in the reservoir reducing from 24.3% to 3.0% (*P* < 0.001) and in rice fields from 16.1% to 1.7% (*P* = 0.005).

### Impact of health education

Health-education campaigns resulted in substantial increases in community awareness about liver fluke transmission and prevention. Awareness levels improved from 45.1% in 2016 to 82.6% in 2022 (*P* < 0.001). These changes were accompanied by a sharp decline in raw fish consumption, from 52.4% in 2016 to 12.3% in 2022 (*P* < 0.001). Correlated with this was a significant reduction in reinfection rates in humans, from 17.4% in 2016 to 9.7% in 2022 (*P* = 0.003) (Table 3). Health-education efforts were reinforced by active participation from 220 health volunteers.

**Table 3:** Intervention strategies and achieved outcomes

Intervention activity/outcomes	Epidemiological study (year 2016)		Sustainability phase (year 2022)		*P*-value
	*n*	Positive/attended (%)	*n*	Positive/attended (%)	
Anthelminthic treatment					
Prevalence of *Opisthorchis* *viverrini* infection in humans	5412	765 (14.1%)	3697	32 (0.9%)	< 0.001^a^
Reinfection of *O*. *viverrini* in humans	655 (from pilot study)	114 (17.4%)	299	29 (9.7%)	0.003^a^
Health education campaigns					
Awareness through health education campaigns	5412	2441 (45.1%)	3697	3054 (82.6%)	< 0.001^a^
Raw fish consumption	5412	2838 (52.4%)	3697	456 (12.3%)	< 0.001^a^
Biological control of *Bithynia* snails					
Reduction in average snail population in owners' swamps	Determined in 100 m^2^ randomly	30 snails/m^2^	determine in 100 m^2^ randomly	< 5 snails/m^2^	< 0.001^b^
Veterinary interventions					
Prevalence of *O. viverrini* infections in dogs and cats	451	96 (21.3%)	320	12 (3.8%)	< 0.001^a^
Construction of toilets in rice fields and near the reservoir					
Consistent use of sanitary toilets	5412	1716 (31.7%)	3697	3220 (87.1%)	< 0.001^a^
Health volunteers’ network					
Households engaged by health volunteers	763	420 (55.1%)	435	409 (94%)	< 0.001^a^

^a^ Chi-squared test comparing the annual proportions of positive cases between the epidemiology and sustainability phases

^b^ Student’s *t*-test comparing the mean infection intensities between the epidemiology and sustainability phases

### Impact of biological and environmental control

Biological-control measures, including the deployment of domestic ducks to reduce *Bithynia* snail populations, were implemented effectively. Snail density in privately owned swamps, ponds and rice fields decreased from 30 snails/m^2^ in 2016 to fewer than 5 snails/m^2^ in 2022 (*P* < 0.001) (Table 3). No significant reduction was observed in the main reservoir (data not shown), possibly due to the abundance of water hyacinth shielding snails from ducks. Periodic assessments showed that snail reduction was consistent across the private water bodies. Consistent use of sanitary toilets increased markedly from 31.7% of those surveyed in 2016 to 87.1% in 2022 (*P* < 0.001).

### Community engagement and sustainability

By 2022, the health volunteer networks had expanded to cover 94% of households, an increase from 55.1% in 2016 (Table 3). Liver fluke-free fish production by local businesses promoted safe practices and boosted community income. One local enterprise generated annual profits of at least THB 100,000. The Food and Drug Administration (FDA) of Thailand confirmed improved hygiene and reduced contamination risks in this registered business. A community learning center, established in 2021, hosted over 250 trainees in three years.

## Discussion

This study reported significant reductions in *O. viverrini* infections in humans, animals, and the environment through a longitudinal One Health intervention program (conducted from 2016 to 2022). Human prevalence decreased from 14.1% to 0.9%, with infection intensity reducing from 76.9 to 25.5 EPG. Reservoir host infections (dogs and cats) declined from 21.3% to 3.8%, while prevalence of metacercariae in cyprinoid fish decreased from 21.9% to 2.2%. Reinfection rates in humans were reduced from 17.4% to 9.7%. Awareness of the risks of liver-fluke transmission rose from 45.1% to 82.6%, and raw fish consumption declined from 21.2% to 0.9%. Biological controls reduced snail densities from 30 to fewer than 5 snails/m^2^ in privately owned swamps, ponds and rice fields, while sanitation improvements increased toilet use from 31.7% to 87.1% among surveyed participants, reducing fecal contamination. Community engagement expanded health volunteer networks, covering 94% of households by 2022. Local enterprises adopted sustainable liver fluke-free fish production and supported the community learning center. This One Health strategy effectively reduced *O. viverrini* infections and fosters sustainable prevention, serving as a replicable model for controlling zoonotic diseases in endemic areas.

The effectiveness of One Health approaches in controlling parasitic diseases emphasizes the critical role of community involvement in sustaining these interventions [9]. A long-term, mixed-methods study in rural Thailand demonstrated the effectiveness of community engagement in controlling opisthorchiasis [11, 12]. Our pilot study around the Huay Luang Reservoir found an *O. viverrini* prevalence of 31.5% during 2014 and 2015 [14]. In the present study, the remarkable reduction in human *O. viverrini* prevalence highlights the success of integrating community engagement with targeted health education. Behavioral changes were central to this achievement [17], as awareness of transmission routes increased, accompanied by a significant decline in raw fish consumption. These outcomes highlight the importance of culturally tailored health education delivered consistently by trusted local figures, such as health volunteers [18]. The expanded health volunteer network reached 94% of households by 2022, fostering community involvement and reinforcing knowledge. This likely contributed to the consistent decline in reinfection rate, which remains a persistent challenge in Thailand [13]. Before implementation, human reinfection rates were consistently high, peaking at 17.4% in 2016 (2014** − **2015 data [14]), likely due to the lack of health education and despite the administration of praziquantel [14]. However, following the intervention implemented in this study, the reinfection rate declined to 9.7% in 2022, along with a reduction in new infection cases. These findings emphasize the role of sustained and localized education in achieving long-term behavioral shifts essential for disrupting the parasite life cycle [19, 20]. School-based health education effectively modifies raw fish consumption behaviors, particularly among younger populations, preventing *O. viverrini* infections [21]. This strategy not only decreases current infection rates but also establishes long-term prevention by fostering healthy habits at an early age. Despite CCA development being linked to long-term risk exposure, our data revealed a gradual annual decline in hospital-reported CCA cases, likely attributable to liver fluke elimination and control efforts in the study area.

The substantial reduction in reservoir host infection prevalence (dogs and cats) highlights the critical role of veterinary interventions in disrupting *O. viverrini* zoonotic transmission [5, 14]. These hosts contribute to environmental contamination by shedding parasite eggs, perpetuating the life cycle and serving as reinfection sources [7]. Dogs frequently scavenge on discarded rotten fish near reservoirs, intensifying transmission risks [14]. Our findings indicate an association between the reduction in *O. viverrini* prevalence in dogs and in humans. However, note that it has been reported that this reservoir host may not contribute significantly to the human-parasite relationship [22]. Combining veterinary interventions with snail control and health education significantly reduced *O. viverrini* prevalence in the Lawa wetland, Khon Kaen, Thailand [9]. Similar results were also observed in Lao PDR, where treating reservoir hosts decreased infection rates of opisthorchiasis and schistosomiasis in animals and humans while reducing environmental contamination [8]. Targeting reservoir hosts and addressing environmental contamination align with One Health principles to provide a sustainable strategy for controlling zoonotic diseases.

The integration of biological and environmental controls was a pivotal aspect of this eco-health strategy. The deployment of domestic ducks to reduce *Bithynia* snail populations led to a significant decline in snail density. This simple biological control mechanism directly targeted the first intermediate host, significantly reducing opportunities for parasite development and transmission [23]. Environmental sanitation improvements further enhanced the program’s effectiveness. The consistent use of sanitary toilets minimized fecal contamination in water bodies, disrupting continuation of the life cycle [7]. The reduction in prevalence of metacercarial infection in fish underscores the synergy between sanitation measures and biological interventions [11]. These findings highlight the importance of combining biological and environmental strategies to effectively disrupt the life cycle of *O. viverrini.*

The integration of community engagement with economic incentives was pivotal in sustaining the program’s long-term impact [24]. We hypothesized that income generation would boost community interest in sustaining prevention efforts and local economic activities, and this proved to be correct. Liver fluke-free fish enterprises and fish-growing businesses increased income and fostered ownership of health interventions. This dual focus demonstrates the effectiveness of integrated interventions for sustainable community resilience. Establishing a community learning center served as a cornerstone of this strategy, providing a dedicated space for education, training, and capacity building to empower local populations with the knowledge and skills necessary for disease prevention [9]. Over three years, the center trained more than 250 attendees, engaging diverse community members and fostering widespread participation in prevention initiatives [11, 25]. Certified fish farmers and fish-product enterprises reported increased income and improved practices, highlighting how public health initiatives can align with economic development goals. This dual benefit not only incentivized compliance with safe fish-production standards but also strengthened community resilience by supporting sustainable livelihoods. Integrating public health strategies with economic and social initiatives, leveraging local leadership and resources, ensures sustainable disease control and enhances public health and community resilience.

Limitations of this study included the exclusion of some residents inside the study area who did not provide consent to participate. These individuals could have continued to harbor and shed parasites, potentially contributing to environmental contamination. Furthermore, some reinfected participants could not be followed up long-term due to migration or career-related constraints, potentially introducing a bias in the assessment of long-term treatment outcomes.

## Conclusions

This study demonstrates the effectiveness of a One Health integrated strategy in significantly reducing *O. viverrini* infections across humans, animals, and the environment in an endemic region. A combination of health education, veterinary care of reservoir hosts, biological controls, and environmental sanitation disrupted the parasite’s life cycle and promoted sustainable prevention. This approach strengthened community engagement and economic development, offering a scalable framework for controlling zoonotic parasites in other regions.

## Supplementary Information

Supplementary material 1: Fig. Photographs depicting activities conducted during this research.

## Data Availability

The datasets used and/or analysed during the current study are available from the corresponding author on reasonable request.

## Abbreviations

CCA: Cholangiocarcinoma
EPG: Eggs per gram
FDA: Food and Drug Administration
One-step FECT: One-step formalin ethyl-acetateconcentration technique
