# Supplementary figures and images for: One Health integrated strategies for sustainable control of Opisthorchis viverrini infections in rural endemic areas of Thailand
Source: Infect Dis Poverty. 2025 Jun 3;14:42. doi: 10.1186/s40249-025-01315-7 (PMC12131461; doi:10.1186/s40249-025-01315-7)

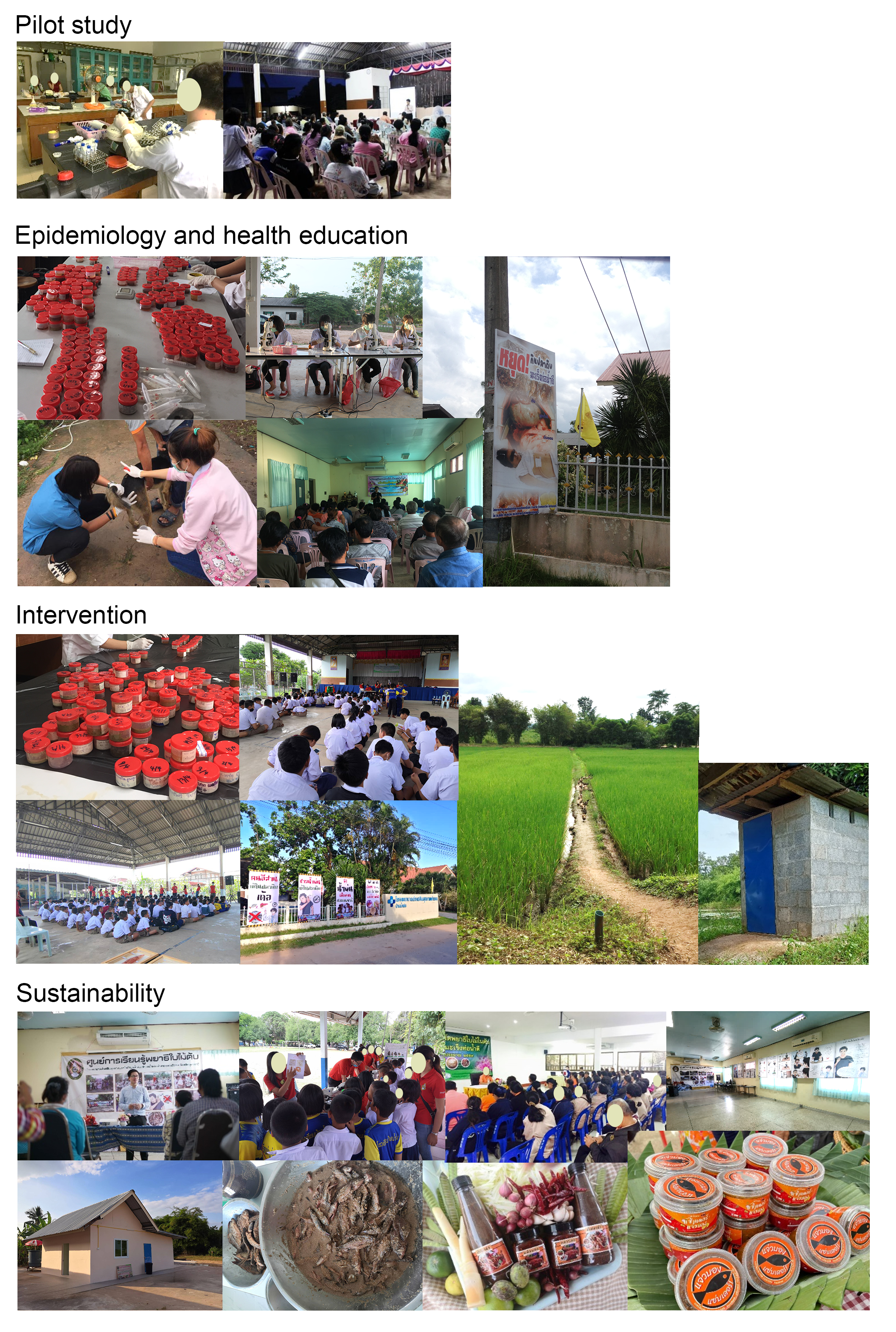

Supplement: Supplementary file 1 — Supplementary material 1: Fig. Photographs depicting activities conducted during this research. [file 40249_2025_1315_MOESM1_ESM.tif]
